# Possibilities of the X-Rays in Surgery

**Published:** 1896-04

**Authors:** 


### Possibilities of the X-Rays in Surgery.

In the first cable dispatch announcing the discovery of Professor Roentgen we were informed that he had succeeded in locating a bullet in the human body by photography, and the surgical side of the discovery, thus early brought to the front, has been kept well in mind and has formed the subject of a good deal of experiments. According to a leading editorial in *The Medical News* of February 22d, surgery has probably gained an aid of real value, although, of course, its powers have been a good deal exaggerated. Says the editorial:

"The wild fancy that this power was a peculiarly penetrating form of 'vision'—a sort of photographic second-sight or 'materialized eye'—which could be caused to calmly penetrate to just such depths of the human tissues as the operator desired and reproduce a complete picture of everything it found at that particular level, is far from the truth. . . . Unfortunately for our antivivisection friends, it must be admitted, also, that the early hope inspired by this power that we should thus be enabled to watch the various functions of the internal organs of the body, and so render vivisection unnecessary, is fast fading into the baseless fabric of a dream.

"The futility of such speculation becomes promptly apparent when we recall the fact that this new picture is not a photograph at all, but simply a shadow or silhouette, so that no details whatever—except as they depend on density and thickness—can be reproduced. . . .

"As far as our present knowledge goes the positive advantages to medicine seem to be limited to three conditions—fracttures, dislocations and tumors of bones, encysted bullets, needles or pieces of glass in the tissues, and earthy calculi. In the first class of conditions its advantages would appear to be slight unless great advances upon present powers and methods can be made. The *tactus eruditus* is certainly a delicate and reliable sense in investigating fractures and dislocations, and it is questionable how much help can be obtained by such crude and blurred shadow pictures as can at present be obtained. In recent cases of fracture or dislocation the delay and discomfort to the patient necessarily involved in the application of the method would be practically an insuperable objection to its use for purposes of diagnosis. . . .

"As a means of ascertaining that the parts had been placed in proper position after adjustment, it would, however, be most valuable, for of course all splints and dressings, except plaster or metallic splints, would be as 'translucent' as the soft tissues themselves. Its principal value, however, would be in obscure cases with much swelling, 'green-stick' fractures for instance, in partial luxations, and in medico-legal cases when the proper setting of a fracture or reduction of a dislocation was in question.

"In the locating of bullets some brilliant results have been already recorded, in which the bullet, beyond the reach of touch or probe, has been found by the X-ray and successfully removed.

"In locating renal and urinary calculi, the Roentgen method will be of great value, although, of course, only as an aid to the sound in the more obscure cases. And Nusser, of Vienna, has already used it with success in demonstrating the presence of a stone in the pelvis of the kidney. It is not yet known whether gall-stones are sufficiently opaque to be detected by its rays."

In conclusion it is suggested that the rays may perhaps prove to have germ-destroying qualities, although the experiments of Dr. Park, of the New York Board of Health, do not bear out this view. The article closes with the following brief notice of these experiments:

"In order to test the influence of the Roentgen rays upon germ life, pure cultures of diphtheria bacilli were subjected to the direct effect of the rays from a Crooke's tube for thirty minutes. Cultures were made both before and after the exposure which were developed in the laboratory by Dr. Park, who reports that no effect whatever was discovered to have resulted from the exposure."

